# Inferring geographic origin in genebank accessions from genome‐wide SNP variation: A barley case study

**DOI:** 10.1002/tpg2.70345

**Published:** 2026-11-13

**Authors:** Stefanie Lück, Martin Mascher, Uwe Scholz, Dimitar K. Douchkov

**Affiliations:** ^1^ Leibniz‐Institut für Pflanzengenetik und Kulturpflanzenforschung (IPK) Seeland Germany

## Abstract

Accurate geographic metadata are essential for using genebank accessions in spatial analyses, but many historical records lack precise coordinates or contain uncertain location information. We developed SNP2GPS, a plant‐genomics workflow for inferring geographic origin from genome‐wide single‐nucleotide polymorphism data and supporting passport metadata curation in barley (*Hordeum vulgare* L.) collections. SNP2GPS integrates genotype preprocessing, neural network‐based coordinate prediction, repeated model training, uncertainty estimation, spatial consistency checks, visualization, and extraction of approximate coordinates from free‐text locality descriptors. Models were trained on curated passport coordinates from the Leibniz Institute of Plant Genetics and Crop Plant Research barley collection and applied to accessions lacking precise geolocation data. On an independent test set of 200 withheld accessions, the base model achieved a mean prediction error of 383 km and a median error of 58 km; an augmented model using additional country‐centroid labels achieved a mean error of 427 km and a median error of 56 km. Country‐centroid augmentation increased geographic coverage in undersampled regions, though at the cost of reduced robustness for some samples. SNP2GPS also identified accessions with large discrepancies between recorded and predicted locations, flagging candidates for metadata review. Prediction uncertainty was lower in wild barley than in domesticated material, consistent with a stronger coupling between genetic structure and geography in wild populations. SNP2GPS provides an open, uncertainty‐aware framework for imputing incomplete geolocation metadata, identifying inconsistent passport records, and delivering reusable predicted coordinates through downloadable outputs and web‐based visualization.

## Plain Language Summary

Genebanks preserve crop diversity for research and breeding, but many accessions lack or have incomplete information about their original sources. This limits the use of these materials in studies of adaptation, diversity, and crop improvement. We developed SNP2GPS, a genomic workflow that uses genome‐wide DNA marker data to infer the likely geographic origin of barley accessions. The method also reports uncertainty, helping users assess the reliability of each prediction. In barley, SNP2GPS predicted geographic origin at the regional scale for many accessions and highlighted records where the recorded and genetically inferred origins strongly disagreed. These cases can guide targeted checks of passport metadata. The predicted coordinates, uncertainty values, software, and web‐based visualizations provide a reusable resource for genebank curation and plant genetic resources research.

**Abbreviations**
API: application programming interface
CPU: central processing unit
GBIS: genebank information system
GBS: genotyping‐by‐sequencing
GPU: graphics processing unit
IPK: Leibniz Institute of Plant Genetics and Crop Plant Research
LLM: large language model
MAF: minor allele frequency
SNP: single‐nucleotide polymorphism

## 1 INTRODUCTION

Genebanks are central infrastructures for conserving and using crop genetic diversity in breeding, adaptation research, and evolutionary studies (Castañeda‐Álvarez et al., 2016; FAO, 2025; Khoury et al., 2010). Their value depends not only on genomic data but also on reliable passport metadata. Geographic origin is particularly important because it allows genetic variation to be interpreted in spatial and environmental context and supports analyses of adaptation, regional diversity, and genotype–environment relationships (Exposito‐Alonso et al., 2018; Forester et al., 2018; Hoban et al., 2016; Rellstab et al., 2015). However, geographic metadata are incomplete or uncertain for many historical genebank records. Accessions may lack precise coordinates and instead be described only by country of origin or free‐text locality information, limiting their use in analyses that require spatial resolution.

Genome‐wide genetic variation often reflects geographic structure shaped by migration, selection, and genetic drift (Bradburd et al., 2016; Novembre et al., 2008; Ralph & Coop, 2013). These patterns can be used to infer geographic origin from genomic data. Previous work established the feasibility of predicting coordinates from genome‐wide variation using deep learning approaches such as Locator (Battey et al., 2020). More recently, Chang and Schmid (2025) applied this concept to barley genebank material and used inferred coordinates in genome–environment association analyses. These studies demonstrate that genomic geolocation can recover useful geographic signals from genetic data.

For genebank applications, however, coordinate prediction alone is not sufficient. Practical metadata curation requires reproducible workflows, uncertainty estimates, quality‐control steps, and interpretable outputs that can help users decide which records require cautious interpretation or manual verification. This is especially important in crop collections, where domestication, breeding, seed exchange, and historical movement can weaken the relationship between genotype and recorded collection site. In such cases, predicted coordinates should be interpreted as estimates of genetic–geographic affinity rather than exact reconstructions of collection sites.

To address these needs, we developed SNP2GPS, a plant‐genomics workflow for inferring geographic origin from genome‐wide single‐nucleotide polymorphism (SNP) data and supporting passport metadata curation in genebank collections. SNP2GPS integrates genotype preprocessing, neural network‐based coordinate prediction, replicated model training, uncertainty‐aware aggregation, spatial consistency checks, visualization, and web‐based access to prediction outputs. The framework is designed to support reproducible analysis of large crop genomic datasets and to make predicted geolocation data accessible for reuse.

In this study, we apply SNP2GPS to a large barley (*Hordeum vulgare* L.) diversity dataset. We evaluate prediction performance using curated passport coordinates and an independent withheld test set, compare a base model with an augmented model that incorporates country‐centroid labels, and examine whether large discrepancies between recorded and predicted locations can flag candidate metadata inconsistencies. We also assess whether prediction uncertainty differs between wild and domesticated barley, reflecting the expected stronger coupling between genetic structure and geography in wild material. By combining genomic prediction with uncertainty reporting, metadata quality control, and accessible visualization, SNP2GPS provides a practical technical advance for improving the usability of genebank genomic resources.

## 2 MATERIALS AND METHODS

### 2.1 Passport data collection and quality control

Passport data, including geographic coordinates, collection‐site information, and country of origin, were obtained from Leibniz Institute of Plant Genetics and Crop Plant Research (IPK) genebank records. The barley passport dataset was exported from the genebank information system (GBIS) and is available as a citable data dump (Lück, 2026a). The barley dataset comprised 22,626 accessions in total, including 2198 accessions classified as wild and 20,428 cultivated/non‐wild accessions. Of these, 2862 accessions had GPS coordinates recorded in the passport data, including 350 wild and 2512 cultivated/non‐wild accessions, whereas 19,764 lacked recorded coordinates, including 1848 wild and 17,916 cultivated/non‐wild accessions. After quality control of the georeferenced records, 2308 accessions were retained for model training, comprising 141 wild and 2167 cultivated/non‐wild accessions.

To improve data consistency, outliers and inconsistent records were identified using a combination of automated and manual filtering steps. First, the Python module global_land_mask (Karin, 2020) was used to verify whether recorded GPS coordinates were located on land, and accessions with coordinates falling in the ocean were excluded. Next, the Python library reverse‐geocode (Penman, 2025) was applied for offline reverse geocoding to infer country names from recorded coordinates. These inferred country names were compared with the reported passport country information, and accessions showing unresolved discrepancies between inferred and reported countries were removed.

To harmonize country information across datasets, the Python package country_converter (Stadler, 2017) was used to standardize country names where possible. Historical or obsolete country designations, including countries or country codes that no longer exist, were manually curated based on the available passport information before consistency checks were finalized. Data processing, filtering, and quality control were implemented programmatically in Pandas (McKinney, 2010). In addition, records sharing identical GPS coordinates but differing in passport collection‐site information were identified and manually resolved. Inspection of the excluded records also revealed recurring spatial artifacts, including grid‐like coordinate patterns in some regions, particularly Iran, suggesting that some reported coordinates represented coarse or systematically assigned locations rather than precise collection sites.

Overall, approximately 19% of the barley accessions with recorded GPS coordinates were excluded because of implausible coordinates, spatial inconsistencies, or unresolved conflicts between geographic and passport information. After preprocessing, only accessions with internally consistent passport metadata were retained for downstream analyses. The geographic distribution of the final curated passport‐coordinate training set is shown in Figure 1a and summarized in Table S1.

**FIGURE 1:**
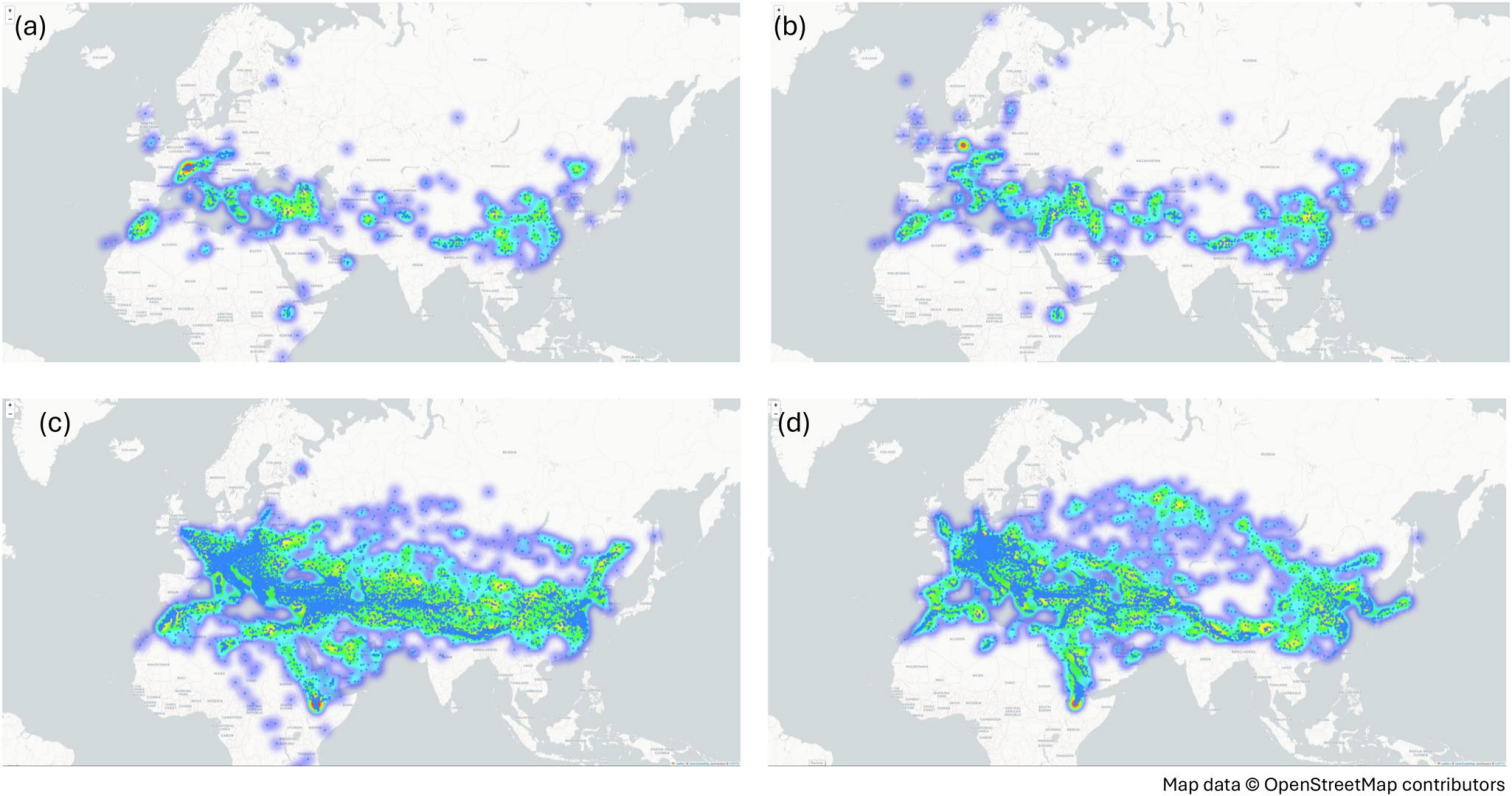
Geographic distribution of training data and SNP2GPS predictions for the barley panel. (a) Curated passport‐based GPS coordinates of barley accessions used for the base training dataset. (b) Augmented training dataset including accessions reassigned to country centroids, resulting in a more clustered country‐level spatial distribution used for model training. (c) Geographic predictions for the barley panel generated by the base SNP2GPS model trained on curated passport coordinates. (d) Geographic predictions for the barley panel generated by the augmented SNP2GPS model trained on the country‐centroid dataset.

### 2.2 Consistency check of passport collection sites and GPS coordinates using a large language model

To further evaluate the consistency between passport collection‐site descriptions and recorded GPS coordinates, we performed an independent text‐based geolocation check using a large language model (LLM). The OpenAI GPT‐4.1 mini model was accessed via the Academic Cloud application programming interface (API) (https://docs.hpc.gwdg.de/services/ai‐services/saia/index.html) and used to infer approximate geographic coordinates from textual descriptions of collection sites in the passport metadata. For each accession, the collection‐site description was provided to the LLM, which returned estimated latitude and longitude coordinates corresponding to the described location.

The LLM‐derived coordinates were then compared with the GPS coordinates recorded in the passport dataset. Geographic distances between both coordinate sets were calculated to quantify agreement between the textual site description and the recorded passport coordinates. Overall, the LLM‐derived coordinates showed strong concordance with the recorded passport data. All but one LLM‐derived location was within 200 km of the corresponding passport coordinates, with a mean distance of approximately 28.75 km (Table S2).

These results indicate that LLM‐based geographic inference can provide useful approximate coordinates from textual descriptions of collection sites, particularly as a consistency check for passport metadata. Manual georeferencing of collection sites from textual descriptions is often time‐consuming, especially for large historical datasets. Automated text‐based geolocation using LLMs may therefore complement traditional curation workflows and help prioritize records for manual inspection when precise GPS data are unavailable or inconsistent.

### 2.3 Generation of an augmented training dataset using country‐centroid coordinates

A major limitation of genebank passport data is the frequent absence of precise geographic coordinates. While some accessions contain recorded GPS coordinates, many records provide only the country of origin. This problem is particularly pronounced for European accessions, where historical collection records often lack detailed geographic coordinates. As a consequence, a large fraction of accessions cannot be used directly for training geolocation prediction models, resulting in sparse training coverage across several important geographic regions.

To increase geographic representation during training, we generated an augmented coordinate dataset for accessions lacking GPS coordinates but having a known country of origin. These accessions were assigned the geographic centroid of the reported country as a coarse proxy coordinate. To limit uncertainty from this procedure, centroid coordinates were assigned only to countries with a total land area smaller than Germany, 357,386 km^2^; larger countries were excluded because one centroid would poorly represent within‐country geographic variation. Country names were standardized using predefined aliases, and each coordinate was annotated by source, original GPS or country centroid. The augmented training set comprised 7663 samples and is summarized in Table S3 and Figure 1b. We then compared a base model trained only on curated GPS coordinates with an augmented model trained on both original and centroid‐derived coordinates.

The centroid imputation procedure was implemented using a custom Python script. The script parsed the passport dataset and standardized country names using a predefined set of aliases to resolve naming inconsistencies. If an accession lacked valid latitude or longitude values but contained a valid country designation, the corresponding country centroid was retrieved and assigned as an approximate geographic coordinate. The origin of each coordinate was recorded in a dedicated metadata field, allowing original GPS coordinates to be distinguished from centroid‐derived values.

To assess the effect of centroid‐based augmentation, we trained and compared two separate SNP2GPS models: a base model using only accessions with original GPS coordinates, and an augmented model incorporating both original and centroid‐derived coordinates.

### 2.4 Genomic data acquisition and preprocessing

To complement the passport metadata, genome‐wide genotyping‐by‐sequencing (GBS) data were retrieved from publicly available resources. The dataset comprised 22,626 barley accessions from the global barley diversity panel described previously by Milner et al. (2019). This dataset provided the genomic basis for training and applying SNP2GPS.

For efficient storage and scalable analysis, the GBS data were converted into Zarr format. Zarr was selected because it provides compression, efficient input/output performance, and access to genotype matrices without requiring the full dataset to be loaded into memory. This structure allows direct chunk‐wise access and is therefore well‐suited for machine‐learning workflows that use batch‐wise data loading.

Prior to model training, several preprocessing steps were applied. Geographic coordinates were normalized to a mean of 0 and a standard deviation of 1 within SNP2GPS to stabilize model training. The software includes optional minor allele frequency (MAF) filtering and basic missing‐value imputation to support direct application to SNP matrices. Missing genotype values were imputed using a binomial sampling approach based on allele frequencies estimated across the dataset. These functions are intended for convenience and to handle residual missing data in already‐processed SNP matrices, not as a substitute for dedicated genotype‐imputation pipelines. For large‐scale, raw, or highly incomplete genotype datasets, we recommend prior preprocessing and imputation using established tools such as BEAGLE, IMPUTE2, or PLINK.

SNPs were filtered using a MAF threshold of 0.01, resulting in approximately 35,006 SNPs. This threshold was selected to reduce feature sparsity, computational cost, and overfitting during neural network training. In exploratory analyses, lowering the threshold to 0.001 did not improve predictive performance but substantially increased computation time; therefore, the 0.01 threshold was retained.

### 2.5 SNP2GPS implementation and model architecture

SNP2GPS was implemented as an open‐source Python pipeline for predicting geographic coordinates from genome‐wide SNP data. The pipeline supports genotype preprocessing, model training, coordinate prediction, aggregation of repeated predictions, and generation of output files for downstream visualization and interpretation (Figure 2). The implementation uses TensorFlow and its high‐level Keras API for neural network training.

**FIGURE 2:**
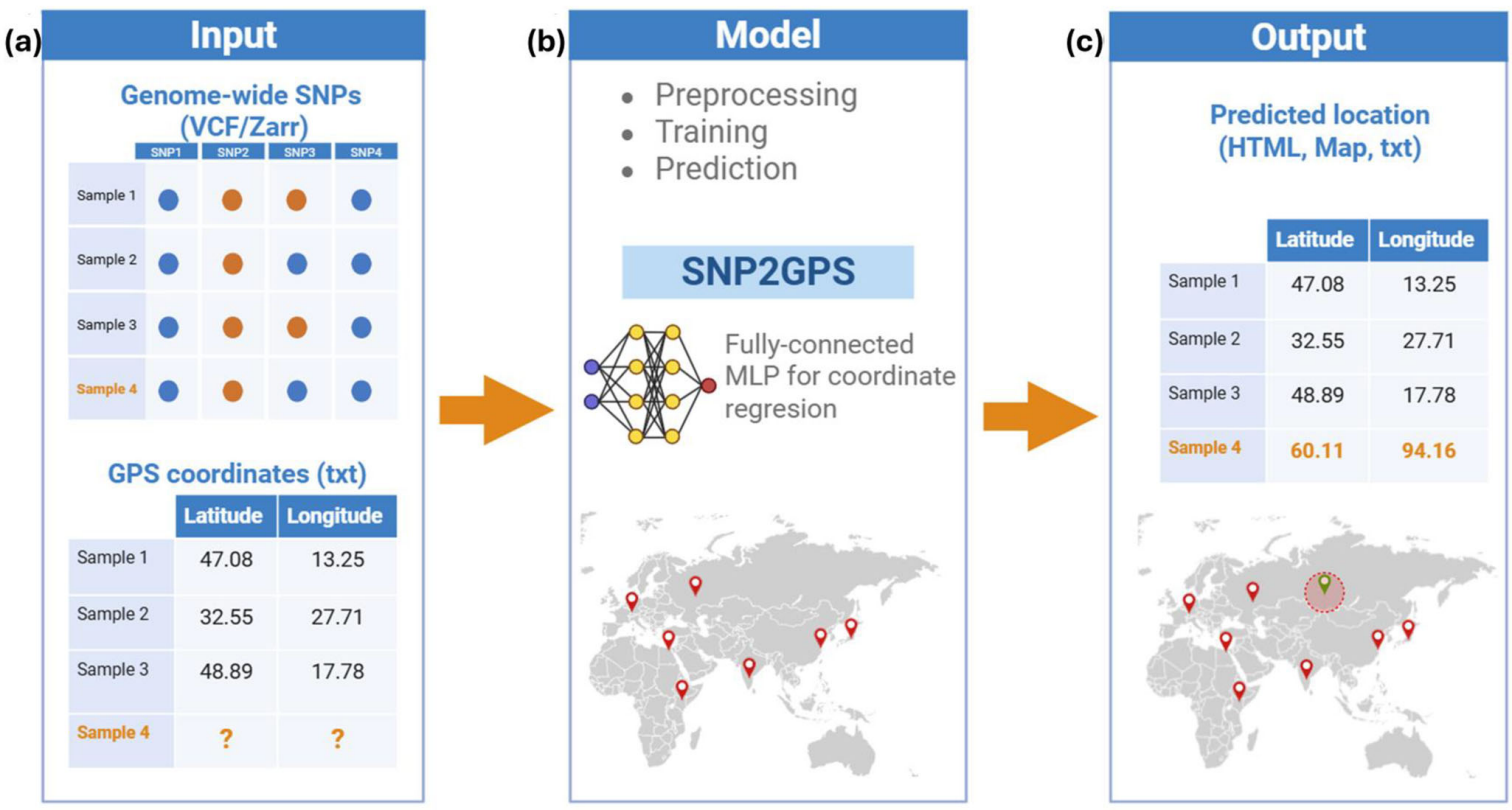
Overview of the SNP2GPS workflow for geographic origin inference from genomic data. (a) Input data consist of a genome‐wide SNP matrix, provided in Variant Call Format (VCF), Zarr, or NumPy format, and corresponding geographic reference metadata containing sample identifiers and coordinates. Accessions with known coordinates are used for model training and validation, whereas accessions lacking coordinates are retained for prediction. (b) SNP2GPS performs preprocessing, neural network training, coordinate prediction, repeated‐run aggregation, outlier filtering, and uncertainty estimation. The predictive model is a configurable, fully connected multilayer perceptron trained to regress latitude and longitude from genome‐wide single‐nucleotide polymorphism (SNP) profiles. (c) Output files include predicted coordinates for accessions lacking passport GPS information, uncertainty metrics across repeated runs, validation statistics for withheld samples, and map‐based visualizations. HTML, HyperText Markup Language.

A fully connected feed‐forward architecture was selected because the prediction task involves regression from fixed numerical SNP vectors to continuous geographic coordinates. This provides a direct, computationally efficient model for the available training data, without requiring the additional data encoding and model adaptation that would be necessary for general‐purpose language‐model architectures. The architecture is configurable via command‐line arguments, including the number of hidden layers, the number of neurons per layer, the dropout rate, the number of training epochs, the batch size, and the number of independent training runs. For the barley analysis, the network contained 10 fully connected hidden layers with 256 neurons per layer. Input features were batch‐normalized before entering the hidden layers, which used exponential linear unit activation functions. A dropout layer with a dropout rate of 0.25 was inserted after the first five hidden layers to reduce overfitting. The output layer consisted of two linear neurons representing the normalized latitude and longitude coordinates.

Model training was performed using the Adam optimizer with Euclidean distance between observed and predicted normalized coordinates as the loss function. Samples with known geographic coordinates were randomly split into training and validation sets at a 75:25 ratio. Models were trained with a batch size of 32 for a maximum of 200 epochs. Validation loss was monitored during training, and early stopping was applied, with the best performing model weights retained. The learning rate was reduced when validation loss ceased to improve.

To assess sensitivity to model architecture and regularization, we evaluated seven hyperparameter configurations varying the number of hidden layers (5, 10, and 15), the number of neurons per layer (128, 256, and 512), and the dropout rate (0.10, 0.25, and 0.50). All configurations were evaluated using the same validation accessions and random seeds, with three independent runs per configuration. Prediction performance was relatively stable across the tested settings, and the architecture used for the main analysis, 10 hidden layers with 256 neurons per layer and a dropout rate of 0.25, showed the lowest mean geographic validation error. Detailed results are provided in Table S4.

For each run, samples with known geographic coordinates were divided into training and validation subsets, using a training proportion of 0.75 unless specified otherwise. The trained model was then used to predict coordinates for withheld validation samples and for accessions lacking GPS information. SNP2GPS supports multiple independent training runs with different random seeds and independent train‐validation splits. Predictions from repeated runs were aggregated after *Z*‐score‐based outlier filtering, using a configurable threshold with a default value of 1.5. Final predicted coordinates were reported as mean latitude and longitude, together with centroid coordinates calculated from the retained predictions. The standard deviation across repeated predictions was reported as an estimate of prediction uncertainty. For the barley analysis, an independent set of 200 accessions with curated passport coordinates was withheld before model training. This set was not used for fitting either the base or augmented model and was used only for final evaluation of prediction accuracy on unseen accessions.

Predictive performance was evaluated using complementary statistical and geographic metrics. Coefficients of determination, *R* ^2^, were calculated separately for latitude and longitude. Geographic prediction error was calculated as the haversine distance between predicted and recorded coordinates and summarized using mean, median, quantiles, and proportions of samples falling below defined distance thresholds. This allowed both overall prediction accuracy and the influence of large outliers to be assessed.

Following prediction aggregation, SNP2GPS also performs a geographic plausibility check of predicted coordinates. Country boundaries were obtained from a global administrative boundary dataset and processed using GeoPandas. For each predicted coordinate, a spatial query was used to determine whether the point was located within a recognized land polygon. If a predicted coordinate was located outside land, for example, in an ocean or large water body, the algorithm searched for the nearest valid land coordinate by evaluating points at increasing distances in eight directions. Corrected coordinates and the corresponding country assignment were stored together with the original prediction output, allowing users to distinguish statistical predictions from geographically adjusted coordinates.

Model training and software testing were performed on a workstation equipped with an NVIDIA RTX 3090 GPU and 128 GB RAM. SNP2GPS can also be executed on CPU (central processing unit)‐only systems, although GPU (graphics processing unit) acceleration is recommended for large datasets and repeated training runs. The source code, documentation, example commands, and parameter descriptions are provided in the project GitHub repository and referenced in the Data Availability Statement section.

### 2.6 Visualization

SNP2GPS generates visual outputs and an integrated HTML (HyperText Markup Language) report to support the interpretation of geographic predictions. The report includes training diagnostics, validation summaries, error‐distance distributions, scatter plots comparing recorded and predicted latitude and longitude, and interactive maps showing reference, predicted, and validation samples. Prediction maps include uncertainty‐based visualization, and land‐correction outputs are summarized where relevant. These outputs provide a reproducible overview of model performance, spatial prediction patterns, and prediction uncertainty.

Static visualizations were generated using Matplotlib (version 3.9.4), and interactive geographic maps were created using Folium (version 0.20.0).

### 2.7 Software installation and usage

For improved reproducibility, SNP2GPS is executed in a dedicated software environment, with conda recommended for installation and dependency management. The software supports both data preprocessing, such as Variant Call Format (VCF)‐to‐Zarr conversion, and the full prediction pipeline, including model training, geographic prediction, visualization, and report generation.

The pipeline requires two input files:

‐ genotypic data in VCF format containing SNP variants and sample identifiers.
‐ geographic reference data in tab‐separated (TSV) format containing three columns: latitude, longitude, and ID.

The ID entries must exactly match the sample identifiers in the VCF file to ensure correct mapping between genomic data and geographic metadata.

To convert a compressed VCF file into Zarr format:

python snp2gps.py convert –vcf example.vcf.gz –zarr example_output.zarr

To run the full SNP2GPS prediction pipeline using a Zarr‐formatted genotype dataset and corresponding geographic metadata:

python snp2gps.py snp2gps –path_zarr example_output.zarr –gps_data example_gps.txt

Additional parameters, such as the number of training epochs, the dropout rate, and the number of independent training runs, can be specified through command‐line arguments to adapt the workflow to different datasets and computational requirements. A complete overview of available parameters, together with usage examples, is provided in the SNP2GPS user manual and software documentation.

All datasets used in this study, along with detailed installation instructions and step‐by‐step guidance for reproducing the results presented in this publication, are available in the project's GitHub repository (Lück, 2026b).

### 2.8 Synthetic dataset for evaluating coordinate‐label inconsistencies

To evaluate whether SNP2GPS can detect coordinate‐label inconsistencies, we generated a synthetic SNP dataset in Python. The dataset comprised 1000 samples and 20,000 SNP features. Samples were assigned to geographically structured clusters, each associated with a predefined geographic center. Individual sample coordinates were drawn from normal distributions around these centers. For each SNP, a global allele frequency was sampled and then modified by cluster‐specific deviations to introduce spatially structured genetic variation. Diploid genotypes were subsequently sampled from binomial distributions using the corresponding cluster‐specific allele frequencies.

To test robustness to coordinate‐label errors, 80 samples, corresponding to 8% of the dataset, were randomly selected, and their latitude and longitude values were exchanged. These swapped cases were kept as a minority class and were selected such that the resulting coordinates remained numerically valid, making them difficult to detect using simple coordinate‐range checks.

The resulting genotype matrix was stored in compressed NumPy format, .npz, in variants‐by‐samples orientation, together with variant and sample identifiers. Sample metadata, including geographic coordinates and swap status, were stored separately. The synthetic dataset was analyzed with the same SNP2GPS workflow used for the barley data, allowing predicted coordinates to be compared with both the original simulated coordinates and the deliberately swapped metadata.

### 2.9 Web portal implementation

A web‐based application was developed using Oracle APEX to provide access to the predicted barley geolocation dataset (Lück, 2026c). The portal was designed to enable querying, filtering, visualization, and download of SNP2GPS outputs. Predicted coordinates, available passport coordinates, prediction uncertainty metrics, and accession‐level metadata were imported into a structured database. Interactive maps were implemented to display predicted and recorded locations, and tabular views support filtering by accession identifiers, geographic regions, and passport metadata. Download functions allow reuse of aggregated prediction outputs in external analyses.

The portal also integrates an LLM‐assisted georeferencing tool for extracting approximate locations from free‐text collection‐site descriptors. This function allows users to submit textual locality information and obtain suggested coordinates that can be compared with existing passport coordinates or SNP2GPS predictions. By combining genomic prediction with artificial intelligence‐based interpretation of unstructured metadata, the portal supports both coordinate inference and practical metadata curation.

## 3 RESULTS

### 3.1 SNP2GPS generated predicted geographic coordinates for the barley panel

We trained two SNP2GPS models for barley: a base model using curated passport GPS coordinates and an augmented model combining curated GPS coordinates with country‐centroid coordinates. Both models were run 100 times with independent random seeds, and accession‐level predictions were aggregated across runs to generate final predicted coordinates and uncertainty estimates. Together, the two models produced downloadable prediction outputs for the barley panel, including mean coordinates, centroid coordinates, and standard deviations across repeated runs (Tables S5 and S6).

In cross‐validation, the base model showed *R* ^2^ values of 0.8833 for latitude and 0.9081 for longitude. The mean geographic validation error was 372.3 km, whereas the median error was 74.4 km (Figure 1c). The augmented model showed *R* ^2^ values of 0.8566 for latitude and 0.8296 for longitude, with a mean validation error of 394.1 km and a median validation error of 50.2 km (Figure 1d). The difference between mean and median errors indicates a skewed error distribution, with many accessions predicted at regional scale and a smaller number showing large deviations. The augmented model increased geographic coverage and showed a slightly lower median error, but also lower longitude *R* ^2^ and a higher mean error than the base model, consistent with the lower spatial precision of centroid‐derived training labels.

### 3.2 Independent test‐set evaluation confirmed regional‐scale prediction accuracy

Both barley models were evaluated using the same independent test set of 200 accessions with curated passport coordinates. These accessions were withheld before model training and were not used to fit either the base or the augmented model. Prediction accuracy was assessed as the geographic distance between recorded passport coordinates and coordinates predicted from GBS data.

The base model showed a mean prediction error of 382.76 km and a median error of 57.53 km. A total of 61.0% of predictions fell within 100 km of the recorded passport coordinates, and 85.0% were within 500 km. However, 10.0% of predictions exceeded 1000 km, with a maximum error of 9175 km, indicating a skewed error distribution with occasional large deviations (Table S7).

The augmented model showed a mean prediction error of 426.96 km and a median error of 55.76 km. A total of 59.0% of predictions fell within 100 km, and 78.0% were within 500 km. In addition, 8.5% of predictions exceeded 1000 km, with a maximum error of 8174 km (Table S8). Summary statistics comparing the base and augmented models on the independent test set are provided in Table S9.

Overall, both models showed comparable median performance, indicating that geographic origin can be inferred at regional scale for many accessions. The base model showed a higher proportion of predictions within 500 km, indicating greater robustness across the independent test set. The augmented model retained similar median accuracy but showed reduced performance at broader error thresholds, consistent with the lower spatial precision of centroid‐derived training labels.

### 3.3 Prediction error and uncertainty differed between biological groups

Geographic prediction error was also compared between wild and cultivated/non‐wild barley. Mean prediction error was numerically lower for wild barley in both the base and augmented models, but the differences were not statistically significant (Table S10).

Prediction uncertainty differed between biological groups. Wild barley accessions showed lower variability among repeated predictions than the remaining accessions, with a median prediction uncertainty of approximately 24 km compared with approximately 54 km for the remaining material. This supports the expectation that genomic geolocation performs best when genetic structure remains closely coupled to geography. In domesticated barley, human‐mediated seed movement, breeding, admixture, growth habit, row type, and end‐use differentiation can weaken the relationship between genotype and recorded collection site, increasing prediction uncertainty.

### 3.4 Large prediction errors flagged candidate passport metadata inconsistencies

Samples with large prediction errors, defined as deviations greater than 1000 km between predicted and recorded passport coordinates, were examined to identify possible causes of discrepancy. Such cases can arise from sparse regional representation in the training data, non‐geographic population structure, historical germplasm movement, admixture, model uncertainty, or inconsistencies in passport metadata. Therefore, large prediction errors were interpreted as signals for further evaluation rather than as simple model failures.

For several high‐error accessions, principal component analysis showed that the samples did not cluster with accessions from the regions indicated by their recorded passport coordinates. For example, BCC648, recorded as originating from Japan, was located within a predominantly European genetic cluster, consistent with its SNP2GPS‐predicted location in Europe (Figure S1). This pattern indicates that, in these cases, the predicted coordinates were more consistent with genome‐wide genetic similarity than with the recorded passport coordinates. Such discrepancies may occur when passport coordinates correctly describe the collection site, whereas genomic similarity reflects a different region of ancestry, diversification, exchange, or cultivation history.

To test whether SNP2GPS can detect a defined coordinate‐label error, we used a controlled synthetic SNP dataset in which a subset of samples was deliberately assigned transposed latitude and longitude values while the underlying genotype–geography structure was retained. SNP2GPS predictions for swapped samples were generally closer to the original simulated coordinates than to the erroneous swapped metadata. For example, sample cleanv2_0022 had swapped recorded coordinates of 57.23, 47.93, whereas its original simulated location was 47.93, 57.23. The model predicted coordinates near 51.55, 58.24, placing the prediction closer to the original location than to the swapped metadata. Similar patterns were observed for additional swapped samples, including cases with large apparent errors relative to the corrupted metadata (Figure 3, Table S11).

**FIGURE 3:**
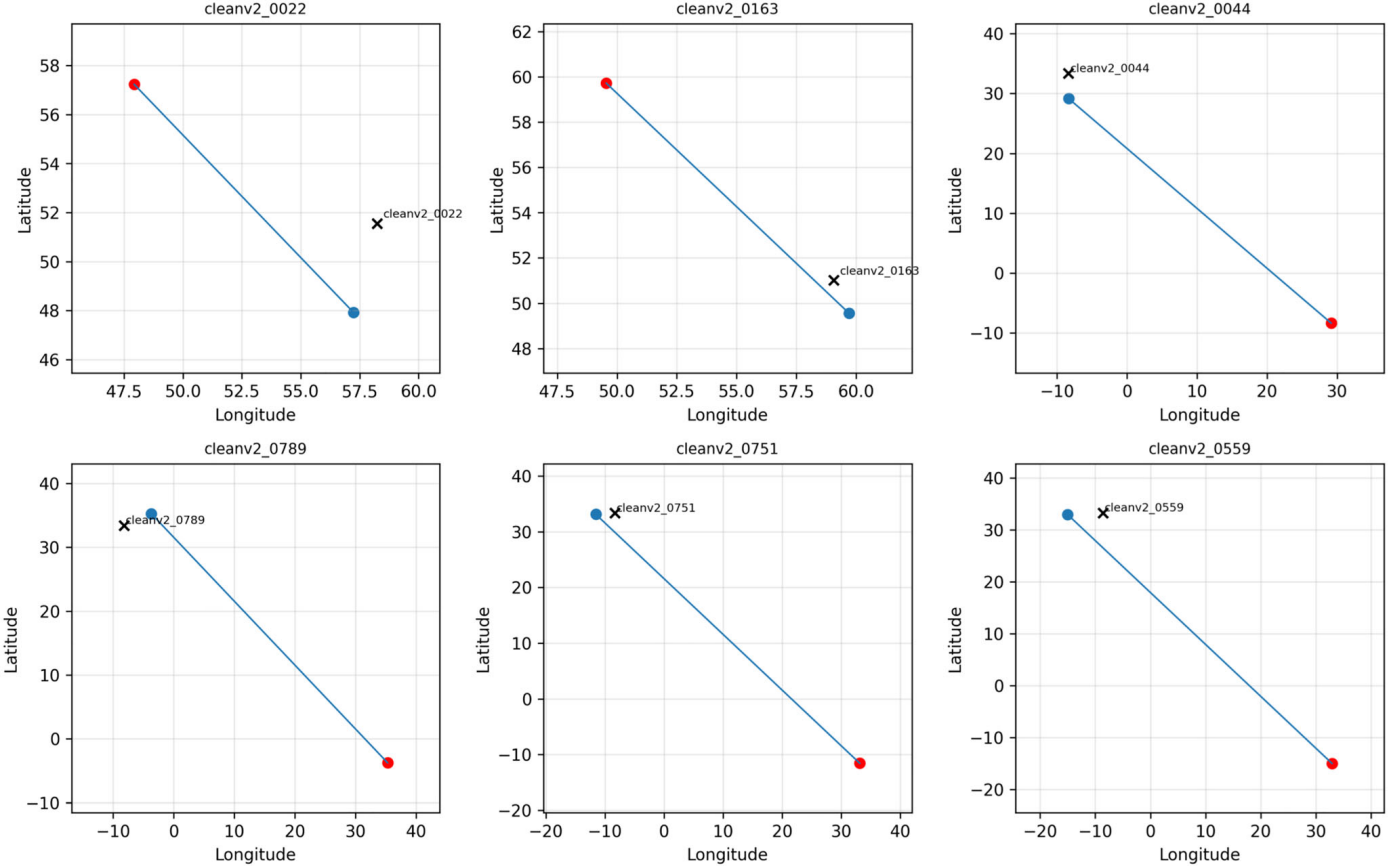
Detection of latitude‐longitude metadata swaps by SNP2GPS. Representative samples from the synthetic dataset with deliberately transposed latitude and longitude coordinates are shown. Blue circles indicate the true simulated locations, red circles indicate the erroneous swapped metadata coordinates, and black crosses indicate SNP2GPS predictions. Connecting lines show the displacement introduced by the latitude‐longitude swap. In most cases, the SNP2GPS predictions are located substantially closer to the true simulated positions than to the erroneous metadata coordinates.

Together, the real‐data examples and the controlled coordinate‐swap experiment show that large discrepancies between predicted and recorded passport coordinates can be informative. SNP2GPS predictions should not be treated as definitive replacements for passport data, but they can prioritize accessions for targeted metadata review, especially when genetic clustering, nearest neighbor relationships, prediction consistency, and a plausible passport‐data error mechanism support large discrepancies.

### 3.5 Spherical encoding improved prediction across the longitude discontinuity

Direct latitude‐longitude regression can be affected by the ±180° longitude discontinuity, where geographically close points on opposite sides of the International Date Line appear numerically distant. This issue is minor for the barley dataset, which is largely confined to Eurasia, but it can affect globally distributed datasets.

SNP2GPS therefore includes an optional spherical target representation through the target_mode parameter. In this mode, latitude and longitude are transformed into three‐dimensional Cartesian coordinates on the unit sphere before model training and converted back after prediction.

In a synthetic dataset near the International Date Line, spherical encoding improved longitude prediction compared with direct latitude‐longitude regression. Longitude *R* ^2^ increased from 0.5745 to 0.6851, mean prediction error decreased from 293 to 236 km, and median prediction error decreased from 256 to 212 km. Thus, direct latitude‐longitude regression was sufficient for the barley dataset, whereas spherical encoding is preferable for datasets affected by longitude discontinuity.

### 3.6 Web portal provides access to predicted barley geolocation data

The SNP2GPS web portal provides interactive access to the predicted geolocation dataset for barley accessions (Figure 4; Lück, 2026c). Users can query accessions by identifier, geographic region, or passport metadata, visualize predicted locations on interactive maps, and compare predictions with recorded passport coordinates where available.

**FIGURE 4:**
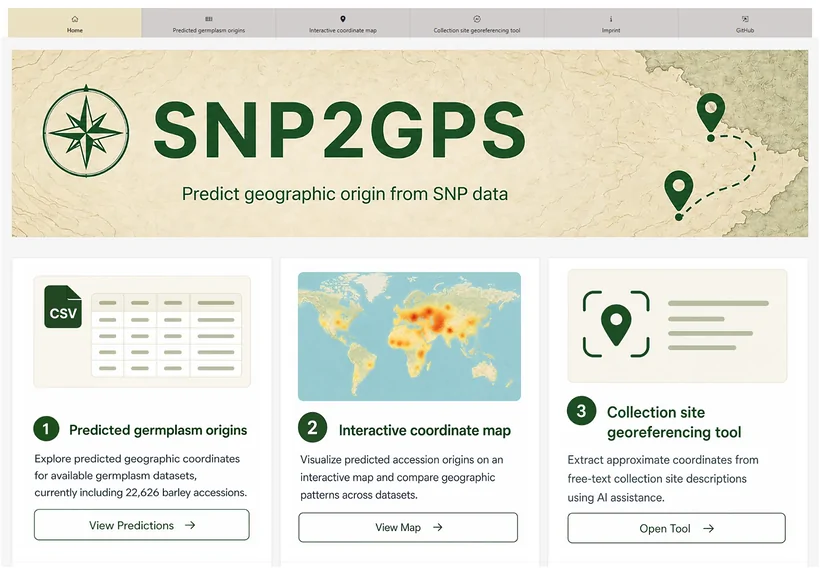
The SNP2GPS web portal provides access to predicted geographic origins inferred from genome‐wide SNP data. The homepage links to three main functions: a searchable table of predicted germplasm origins, currently including 22,626 barley accessions; an interactive coordinate map for visualizing predicted accession origins and geographic patterns; and an AI‐assisted collection site georeferencing tool for extracting approximate coordinates from free‐text locality descriptions. Additional links provide access to project information, imprint, and the SNP2GPS GitHub repository.

The portal provides access to aggregated prediction outputs, including mean coordinates, centroid positions, and standard deviations across repeated model runs. These uncertainty metrics allow users to distinguish accessions with stable predictions from cases requiring cautious interpretation or further metadata inspection. Download options enable reuse of the dataset in external analyses.

The portal also includes an LLM‐assisted georeferencing tool for extracting approximate coordinates from free‐text locality descriptions. By combining accession‐level filtering, map‐based visualization, uncertainty metrics, downloadable outputs, and text‐based georeferencing, the portal makes SNP2GPS predictions accessible as a reusable genebank metadata resource.

### 3.7 SNP2GPS showed comparable performance to Locator on a reference dataset

To compare SNP2GPS with an established genomic geolocation method, we evaluated it on the publicly available test dataset provided with Locator. This comparison was used to determine whether SNP2GPS produced prediction results within the performance range of the reference implementation when applied to the same input data.

The two implementations produced broadly similar results, although prediction error is reported differently. Locator reports a mean validation error of 3.76 and a median error of 3.30, calculated as Euclidean distance in latitude‐longitude space. These values are expressed in degrees and therefore do not directly represent physical distances on the Earth's surface. SNP2GPS reports geographic error as haversine distance, resulting in a mean validation error of 429.66 km and a median error of 350.14 km. Using an approximate conversion of 111 km per degree, the Locator errors correspond to approximately 417 and 366 km, respectively.

Similar *R* ^2^ values were obtained for both implementations, supporting comparable coordinate prediction performance on this reference dataset. Runtime was also similar under the tested conditions, with Locator requiring approximately 0.402 s and SNP2GPS approximately 0.43 s (Table S12).

This comparison indicates that SNP2GPS reproduces Locator's performance range while extending the workflow toward genebank‐oriented applications, including repeated‐run aggregation, uncertainty reporting, metadata inconsistency flagging, spatial plausibility checks, integrated visualization, and web‐based access to prediction outputs.

## 4 DISCUSSION

Accurate geographic metadata are important for interpreting genebank accessions, but many historical records lack precise coordinates or contain uncertain location information. SNP2GPS addresses this problem as a plant‐genomics workflow for inferring geographic origin from genome‐wide SNP data and for supporting passport metadata curation. Its main contribution is not only coordinate prediction, but the integration of repeated model training, uncertainty‐aware aggregation, metadata inconsistency flagging, spatial plausibility checks, visualization, and web‐based access to prediction outputs.

Previous work established that geographic coordinates can be inferred from genome‐wide variation using deep learning approaches, including Locator (Battey et al., 2020), and this concept has recently been applied to barley genebank material (Chang & Schmid, 2025). Using a georeferenced barley landrace dataset filtered with GGoutlieR, Chang and Schmid (2025) reported *R* ^2^ values of 0.987 for longitude and 0.921 for latitude, compared with 0.908 and 0.883, respectively, for the base SNP2GPS model. These values should not be interpreted as a direct performance comparison, as the studies differ in accession composition, marker filtering, data quality control, and validation design. SNP2GPS builds on this general framework but addresses a different practical objective: supporting genebank metadata workflows. The comparison with Locator showed broadly comparable prediction performance on the reference dataset, with differences in reported error values largely explained by the use of different distance metrics and units. The added value of SNP2GPS is therefore not a claim of superior coordinate prediction, but the combination of prediction, uncertainty reporting, quality control, and accessible outputs in a workflow tailored to large crop collections.

In barley, SNP2GPS inferred geographic origin with useful regional‐scale accuracy for many accessions. The base model showed a median independent test‐set error of 57.53 km, while the augmented model showed a comparable median error of 55.76 km. However, mean errors were substantially higher than median errors, indicating skewed error distributions with a minority of large deviations. This pattern is important for practical use. Predicted coordinates should be interpreted as estimates of geographic origin or genetic‐geographic affinity, not as exact reconstructions of collection sites.

The comparison between the base and augmented models illustrates a trade‐off between spatial precision and geographic coverage. The augmented model increased the number and geographic distribution of training samples by incorporating country‐centroid labels and retained similar median accuracy, but showed reduced performance at broader error thresholds. Because multiple accessions from the same country were assigned identical centroid coordinates, this augmentation also concentrated training labels at discrete geographic locations and changed the spatial weighting of the training set. This effect likely contributed to the regional differences observed between the prediction maps of the base and augmented models. Country‐centroid augmentation can therefore be useful for expanding representation in undersampled regions, but it should be treated as a coarse‐label strategy rather than a replacement for precise passport coordinates.

A key factor affecting prediction reliability is the strength of the relationship between genetic structure and geography. Wild barley accessions showed lower prediction uncertainty than the remaining material, consistent with a stronger coupling between ancestry and geographic origin in wild populations. In domesticated barley, this relationship can be weakened by human‐mediated seed movement, admixture, breeding history, growth habit, row type, end use, and other forms of non‐geographic population structure. These factors may contribute to higher prediction uncertainty in domesticated material, although the observed difference in mean geographic prediction error between wild and cultivated/non‐wild barley was not statistically significant.

Large discrepancies between predicted and recorded passport coordinates can nevertheless be informative. In several high‐error cases, genetic clustering supported regions closer to the SNP2GPS predictions than to the recorded passport coordinates. The synthetic coordinate‐swap experiment further showed that, when sufficient correct training information is available, SNP2GPS can recover the underlying genotype–geography structure despite a limited number of erroneous coordinate labels. These results support the use of large prediction errors as prioritization signals for metadata review. The metadata‐based quality control used here differs conceptually from dedicated geo‐genetic outlier approaches such as GGoutlieR, which identify unusual relationships between genetic and geographic similarity (Chang & Schmid, 2023). However, such cases require careful interpretation because discrepancies may reflect metadata errors, historical movement, sparse training data, non‐geographic population structure, or genuine model limitations. Regional differences in prediction accuracy may therefore arise from unequal reference‐sample density, variation in the strength of local genotype–geography relationships, historical germplasm movement, and differences in the precision of passport coordinates.

The LLM‐assisted georeferencing component provides a complementary route for working with incomplete passport metadata. While SNP2GPS infers geographic origin from genome‐wide variation, the text‐based module extracts approximate coordinates from free‐text locality descriptions. This is useful for historical records where locality information exists, but structured coordinates are missing or uncertain. The approach is not a substitute for expert curation, especially where place names are ambiguous, historical, or duplicated across regions, but it can help prioritize records for manual inspection and improve the usability of incomplete metadata at scale.

Several limitations should be considered. Prediction accuracy depends on the geographic and genetic coverage of the training data, and poorly represented regions remain difficult to resolve. Centroid‐derived coordinates increase training coverage but introduce spatially coarse labels. The method assumes that genome‐wide variation retains a geographic signal, which is more reliable in wild or less redistributed material than in domesticated accessions with complex histories of movement and selection. Predicted coordinates should therefore be interpreted together with uncertainty estimates, genetic clustering, nearest neighbor information, and passport metadata, rather than used as unqualified replacements for recorded coordinates.

Future improvements should focus on expanding well‐curated reference datasets, especially for undersampled regions and wild relatives, and on improving automated detection of metadata inconsistencies. Additional analyses could evaluate how accession type, biological subgroup, and non‐geographic population structure affect prediction error. Further integration of genomic prediction, text‐based georeferencing, and GBISs could strengthen metadata curation workflows and broaden the use of genomic resources in plant genetic resources research.

## 5 CONCLUSION

SNP2GPS demonstrates that genome‐wide SNP data can be used to infer geographic origin in genebank accessions at regional scale and to support the curation of incomplete or inconsistent passport metadata. In barley, repeated model training and aggregation produced stable predictions for many accessions, while uncertainty estimates and large‐error cases provided useful indicators for cautious interpretation and targeted metadata review. The lower prediction uncertainty observed in wild barley supports the expectation that genomic geolocation is most reliable when genetic structure remains closely linked to geography. Beyond coordinate prediction, SNP2GPS provides an integrated framework for uncertainty‐aware inference, detection of candidate metadata inconsistencies, and accessible data delivery through downloadable outputs and web‐based visualization. Used as a decision‐support tool rather than a replacement for passport data, SNP2GPS can improve the usability of genebank genomic resources for diversity analysis, conservation, and crop improvement.

## AUTHOR CONTRIBUTIONS

**Stefanie Lück**: Conceptualization; data curation; formal analysis; investigation; methodology; software; validation; visualization; writing—original draft; writing—review and editing. **Martin Mascher**: Conceptualization; funding acquisition; resources; writing—review and editing. **Uwe Scholz**: Conceptualization; project administration; resources; supervision; writing—review and editing. **Dimitar K. Douchkov**: Conceptualization; funding acquisition; methodology; project administration; resources; supervision; writing—original draft; writing—review and editing.

## CONFLICT OF INTEREST STATEMENT

The authors declare no conflicts of interest.

## Supporting information



Supplemental Figure S1 provides the PCA analysis of the high‐error accession BCC648 and its local genetic neighborhood.

Supplemental Tables S1 to S12 are provided as one combined Excel file. The tables include curated passport‐coordinate, LLM‐derived geolocation checks, the country‐centroid augmented training dataset, model predictions and validation results, comparisons with Locator, synthetic coordinate‐swap validation data, comparisons of prediction error between wild and cultivated/non‐wild barley, and hyperparameter sensitivity analysis.

## Data Availability

The datasets underlying this article are deposited in Zenodo and are accessible at: https://doi.org/10.5281/zenodo.20034272. The SNP2GPS software, documentation, and usage examples are available from the project GitHub repository: https://github.com/snowformatics/SNP2GPS. Predicted barley geolocation data can also be queried and visualized through the SNP2GPS web portal: https://snp2gps.ipk‐gatersleben.de.
