# Supplementary material for: Inferring geographic origin in genebank accessions from genome‐wide SNP variation: A barley case study
Source: Plant Genome. 2026 Nov 13;19(4):e70345. doi: 10.1002/tpg2.70345 (PMC13760490; doi:10.1002/tpg2.70345)
Supplement: Supplementary file 1 [file TPG2-19-e70345-s001.pdf]

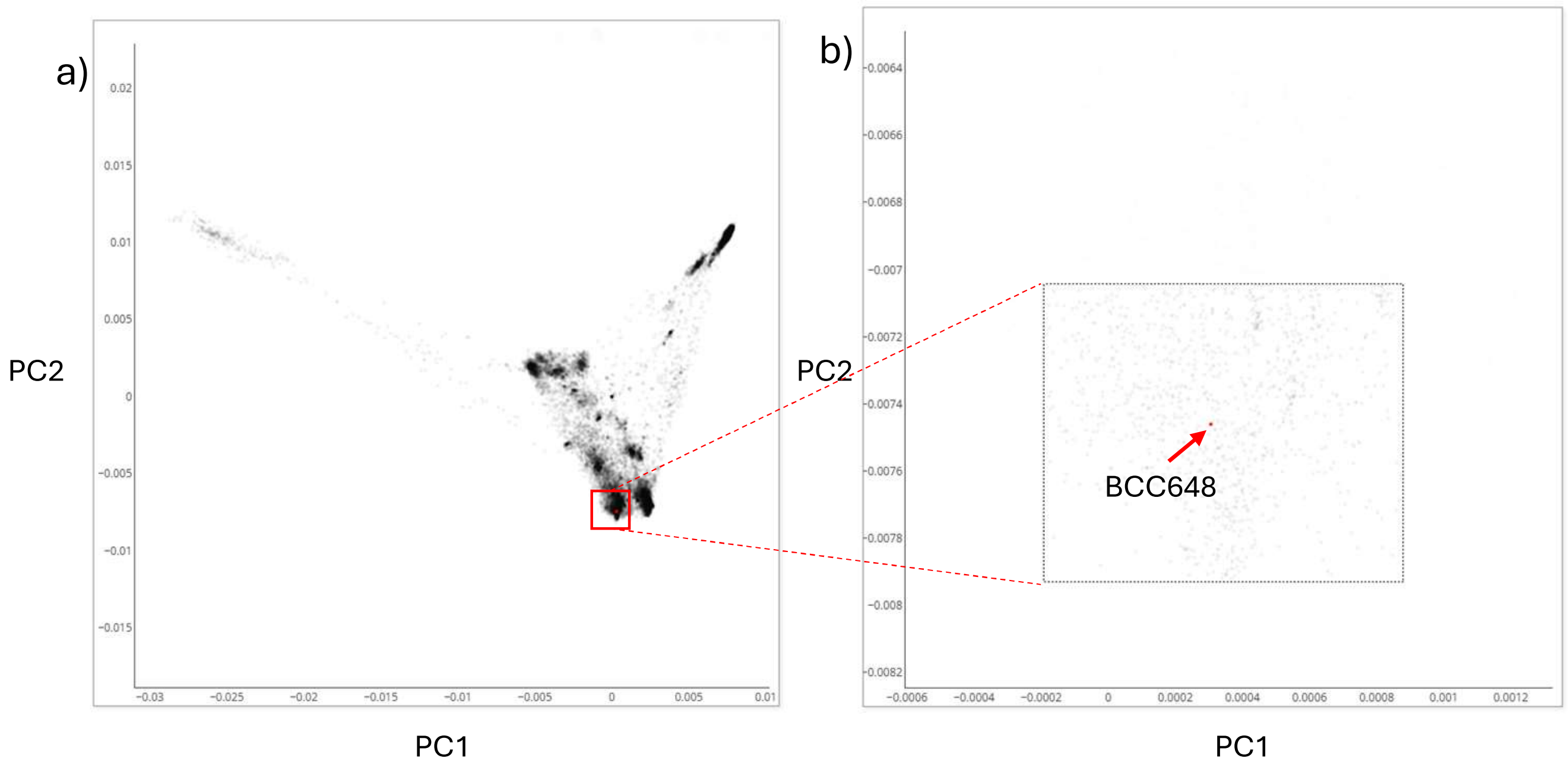

c)

Country	Count	Percent
Germany	249	23.42%
Unknown (not in passport data)	146	13.73%
France	64	6.02%
Germany	50	4.70%
Bulgaria	47	4.42%
Romania	43	4.05%
United States of America	41	3.86%
Austria	36	3.39%
Ukraine	34	3.20%
Hungary	28	2.63%
Poland	28	2.63%
Netherlands	25	2.35%
UdSSR (now: Russian Federation)	25	2.35%
Russian Federation	25	2.35%
Yugoslavia	19	1.79%
Turkey	16	1.51%
Afghanistan	15	1.41%
India	13	1.22%
Switzerland	13	1.22%
Spain	13	1.22%
Czechoslovakia (formerly)	13	1.22%
Italy	13	1.22%
Ethiopia	12	1.13%
Sweden	12	1.13%
Great Britain	9	0.85%
German Democratic Republic (GDR)	9	0.85%
Belgium	8	0.75%
Pakistan	6	0.56%
Iran (Islamic Republic Of)	5	0.47%
China	5	0.47%
Algeria	4	0.38%
Syrian Arab Republic	4	0.38%
Albania	4	0.38%
Canada	3	0.28%
Colombia	3	0.28%
Denmark	2	0.19%
Uzbekistan	2	0.19%
Nepal	2	0.19%
**Japan**	**2**	**0.19%**

Supplemental Figure S1. Principal component analysis of the barley diversity panel highlighting the high-error accession BCC648. (a) PCA based on genome-wide SNP data, with BCC648 highlighted. (b) Enlarged view of the PCA region surrounding BCC648. (c) Country-of-origin composition of accessions within the selected PCA neighborhood around BCC648. BCC648 is recorded as originating from Japan but is located within a predominantly European genetic cluster; only two accessions (0.19%) within the selected neighborhood were recorded as originating from Japan. This genetic placement is consistent with the geographic region predicted by SNP2GPS.
